# Workers’ whole day workload and next day cognitive performance

**DOI:** 10.1007/s12144-023-04400-y

**Published:** 2023-03-03

**Authors:** Raymond Hernandez, Haomiao Jin, Elizabeth A. Pyatak, Shawn C. Roll, Stefan Schneider

**Affiliations:** 1 Dornsife Center for Economic & Social Research, University of Southern California, 90089 Los Angeles, CA USA; 2 Chan Division of Occupational Science and Occupational Therapy, University of Southern California, 90089 Los Angeles, CA USA; 3 School of Health Sciences, University of Surrey, GU2 7YH Guildford, UK; 4 USC Center for Economic & Social Research, 635 Downey Way, VPD 405 Los Angeles, CA USA

**Keywords:** Whole day workload, Sustained attention, Processing speed, Cognitive performance, Type 1 diabetes

## Abstract

Workload experienced over the whole day, not just work periods, may impact worker cognitive performance. We hypothesized that experiencing greater than typical whole day workload would be associated with lower visual processing speed and lower sustained attention ability, on the next day. To test this, we used dynamic structural equation modeling to analyze data from 56 workers with type 1 diabetes. For a two-week period, on smartphones they answered questions about whole day workload at the end of each day, and completed cognitive tests 5 or 6 times throughout each day. Repeated smartphone cognitive tests were used, instead of traditional one- time cognitive assessment in the laboratory, to increase the ecological validity of the cognitive tests. Examples of reported occupations in our sample included housekeeper, teacher, physician, and cashier. On workdays, the mean number of work hours reported was 6.58 (SD 3.5). At the within-person level, greater whole day workload predicted decreased mean processing speed the next day (standardized estimate=-0.10, 95% CI -0.18 to -0.01) using a random intercept model; the relationship was not significant and only demonstrated a tendency toward the expected effect (standardized estimate= -0.07, 95% CI -0.15 to 0.01) in a model with a random intercept and a random regression slope. Whole day workload was not found to be associated with next-day mean sustained attention ability. Study results suggested that just one day of greater than average workload could impact next day processing speed, but future studies with larger sample sizes are needed to corroborate this finding.

## Introduction

Workload is an often-studied construct because of its wide applicability and relationship with important outcomes such as well-being and job performance. Although there is no universally accepted definition of workload (Hart, 2006), excessive workload has been consistently associated with negative outcomes. In meta-analytic reviews, (Bowling & Kirkendall, 2012) and (Bowling et al., 2015) showed that excessive workload is negatively associated with psychological and physical well-being and positively associated with turnover intention and absenteeism. In this article, workload is defined as the cost (e.g. fatigue, stress, illness) of performing tasks, which is consistent with the widely-used and well-validated National Aeronautics and Space Administration Task Load Index (NASA-TLX) (Hart, 2006). Excessive workload measured by NASA-TLX has been shown to relate to increased incidence of burnout (Ziaei et al., 2015), more task errors (Mazur et al., 2012), and greater fatigue (Arellano et al., 2015).

Existing studies have mostly examined workload relevant to work tasks (Charles & Nixon, 2019; Inegbedion et al., 2020), but investigation of workload experienced over entire days (i.e. inclusive of work and non-work) may also be beneficial because of the more holistic perspective provided by the assessment of whole day workload (Hernandez et al., 2021a, b). The National Institute for Occupational Safety and Health (NIOSH) has supported considering both work and non-work factors that may impact worker well-being, acknowledging the close relations between work and non-work experiences (Chari et al., 2018). Furthermore, experiences in and outside of work have been shown to both impact well-being outcomes such as overall stress (Sauter, 2013). Using a whole day workload measure in research echoes NIOSH’s advocacy for a more holistic perspective of worker well-being.

Whole day workload may be important for its potential impact on cognitive functioning, an essential component of work performance. According to the Effort Recovery Model, exerting effort (i.e. exposure to workload) leads to load reactions, acute changes to psychobiological systems that aid in fulfilling task demands in the short term, such as by increasing alertness (Meijman & Mulder, 1998). These load reactions are reversed only with adequate time for recovery, or return of psychobiological systems to baseline, after exertion ceases (Meijman & Mulder, 1998). After sufficient recovery, fatigue and other manifestations of extended exposure to load reactions are reduced (Sonnentag, 2001). However, if recovery is insufficient (Sonnentag, 2001), reduced well-being and efficiency may result, which can manifest as cognitive performance decrements. Greater whole day workload has been found to be associated with decreased frequency of engagement in recovery activities (Hernandez, Pyatak, et al., 2021), so we expected greater whole day workload to be associated with reduced subsequent cognitive performance. Consistent with this, a prior study found that longer work shifts were associated with reduced cognitive performance afterwards (Macdonald & Bendak, 2000). On the other hand, prior work examining the within-person correlations between whole day workload and next day cognitive performance showed no significant associations (Hernandez et al., 2021a, b). This null finding, however, may have been due to not considering improvements in cognitive testing performance over time attributable to practice, which can lead to false conclusions (Bartels et al., 2010).

Workers with chronic conditions may be a population for whom a possible association between whole day workload and cognitive performance may be especially relevant, due to high levels of workload they often experience. In addition to demands from work, workers with chronic conditions must also deal with “patient work”, or health management related activities (Valdez et al., 2015). For instance, health management tasks for individuals with hypertension often includes regular monitoring of blood pressure, taking medications as prescribed, monitoring symptoms (e.g. headaches, shortness of breath), and taking the appropriate corresponding actions as needed (e.g. use of diuretics) (Riegel et al., 2017). The combined demands from work and health management may increase the risk of absenteeism and presenteeism (Jinnett et al., 2017). Greater overall demands may also mean greater likelihood of insufficient recovery in workers with chronic conditions, and corresponding decreased cognitive performance.

### Present study

We examined the relationship between whole day workload and cognitive performance using data from an ecological momentary assessment (EMA) study on workers with type 1 diabetes (T1D). EMA is repeated measurement of people’s momentary experiences in their natural environments (Shiffman et al., 2008). T1D is a condition characterized by beta cell destruction leading to absolute insulin deficiency (Kerner & Brückel, 2014). Unlike type 2 diabetes, T1D is caused by an autoimmune reaction and not lifestyle factors, and age of onset is usually younger (i.e. 20 years of age or less) (Ozougwu, 2013). Patient work for T1D includes self-monitoring of blood glucose, managing regular insulin intake, and problem solving issues that may arise with equipment used (e.g. insulin pump) (Beck et al., 2017). Combined demands from work and T1D health management can at times be problematic (Hansen et al., 2018). The T1D EMA study from which we analyzed data involved the completion of 5 to 6 phone-based surveys per day, for approximately two weeks. Cognitive tests were administered at the end of each survey, and whole day workload was assessed at the end of each day.

In the EMA study analyzed here, the mobile cognitive tests assessed processing speed and sustained attention ability, which are aspects of cognitive functioning relevant to a variety of jobs. In positions such as nursing (Allan et al., 2014) and air traffic control (Hedayati et al., 2021), transient cognitive decrements in these areas may lead not only to decreased work performance, but to safety issues. The dynamic day-to-day relationships between whole day workload and cognition may best be investigated using ambulatory assessments, that is, methods to study people in their natural environments (Trull & Ebner-Priemer, 2013). Cognitive performance and workload have typically been measured on a single occasion (Sliwinski et al., 2018). With one time measurements, however, day to day variability in cognitive performance cannot be captured and, thus, questions about factors impacting this variability cannot be investigated (Bolger et al., 2003). Ambulatory assessments not only capture intra-person variability, but measures taken may also be more ecologically valid as compared to assessment in the laboratory or clinic. Recent work has demonstrated the validity of assessments of cognitive performance and workload in the ambulatory context (Hernandez et al., 2021a, b; Sliwinski et al., 2018), and we take advantage of these ambulatory measures to address our research questions.

Because we analyzed data from a repeated measures EMA study, the relationship between whole day workload and cognitive performance could be conceptualized at both the average (between-person) and momentary (within-person) levels. At the between-person level, experience of higher amounts of workload on average may be associated with decreased frequency of participation in recovery activities (Hernandez, Pyatak, et al., 2021). We therefore expected a higher average level of experienced workload to be associated with decreased average sustained attention ability and processing speed.

At the within-person level, consistent with results from a prior study (Hernandez, Pyatak, et al., 2021), we assumed that higher workload compared to one’s average would imply both greater expenditure of effort and less time for recovery. Accordingly, we hypothesized that greater than average workload on a given day would predict decreased processing speed and less sustained attention ability on the next day, as assessed with standardized ambulatory tests. Examination of same day comparisons between average cognitive performance over a day and workload would not have allowed us to investigate our theory that greater workload (insufficient recovery) would precede decreased cognitive performance, because the whole day workload ratings preceded only the last cognitive test of the day. Acute workload exposure was operationalized as the experience of workload on the day prior to the assessment of cognitive performance because it was a period of workload exposure looked at in prior studies (albeit in work contexts) (Ansiau et al., 2008; Kerstin et al., 2012; Macdonald & Bendak, 2000), that preceded all the cognitive tests taken during the following day. Relationships between average cognitive performance over a day and workload on the same day were still examined however in preliminary analyses.

In terms of additional within-person level tests, we hypothesized that workload would predict worse next day cognitive abilities *after* controlling for sleep quality. Sleep is a recovery event, nestled in time between the experience of whole day workload and the cognitive testing over the following day, that has also been found to affect next day cognition (Kaliyaperumal et al., 2017). By statistically controlling for sleep quality, we ensured that any observed effects of workload on cognition were not attributable to effects of workload on sleep quality. As an exploratory question, we also examined whether higher workload would be associated with slower response times in people’s responses to next-day’s EMA questionnaires. There has been recent evidence that questionnaire response times may reliably approximate momentary cognitive functioning (Roque et al., 2020).

## Methods

### Study overview

Participants were adults 18 years or older, self-identified as workers and recruited using patient lists from three clinical sites as a part of the Function and Emotion in Everyday Life with Type 1 Diabetes (FEEL-T1D) study (Pyatak et al., 2021). After enrollment, participants completed a baseline training call and surveys, two weeks of EMA surveys (5–6 per day) and ambulatory cognitive assessments on a mobile device, wearing of passive sensors (e.g. blood glucose monitor), and a follow-up call with surveys. One phone based cognitive test for processing speed and another for sustained attention were completed after each EMA survey. The first EMA surveys of the day were administered at participant chosen times near their regular waking time, and then at three-hour intervals afterwards. Data collection procedures were approved by the University of Southern California’s Institutional Review Board, and all participants provided informed consent prior to engaging in study procedures.

### Measures

The Mobile EMA application (mEMA: ilumivu.com) was used to administer EMA surveys to participants 5–6 times per day, for approximately two weeks. At each time point, participants completed two cognitive tests after momentary survey items were completed, and their response times for each survey question was recorded. At the last assessment of each day, participants provided a subjective assessment of workload experienced over the whole day. Sleep quality was assessed in the first assessment of each day (Åkerstedt et al., 2002). The table in Appendix A lists all study measures used in analyses. The full list of the items administered is outlined in (Pyatak et al., 2021).

#### Workload

Whole day workload was assessed using a version of the National Aeronautics and Space Administration Task Load Index (NASA-TLX) (Hart & Staveland, 1988) adapted for use in the whole day EMA context, with preliminary evidence supporting its validity (Hernandez et al., 2021a, b). At the end of a day, participants are asked to provide ratings of the mental demand, physical demand, temporal demand, performance, effort, and frustration level experienced over the day, all on scales of 0 to 100. These ratings were averaged to create a summary score of whole day workload.

#### Sleep quality

A single item (“how rested did you feel”) was used to assess sleep quality each morning. This item has been used as an indicator of sleep quality in prior research (Åkerstedt et al., 2002; Galinsky et al., 2018; Keklund & Åkerstedt, 1997), though most often alongside other indicators. To minimize burden in the EMA context, the “rested” item was the only sleep quality question used.

#### Cognitive functioning measures

**Fig. 1:**
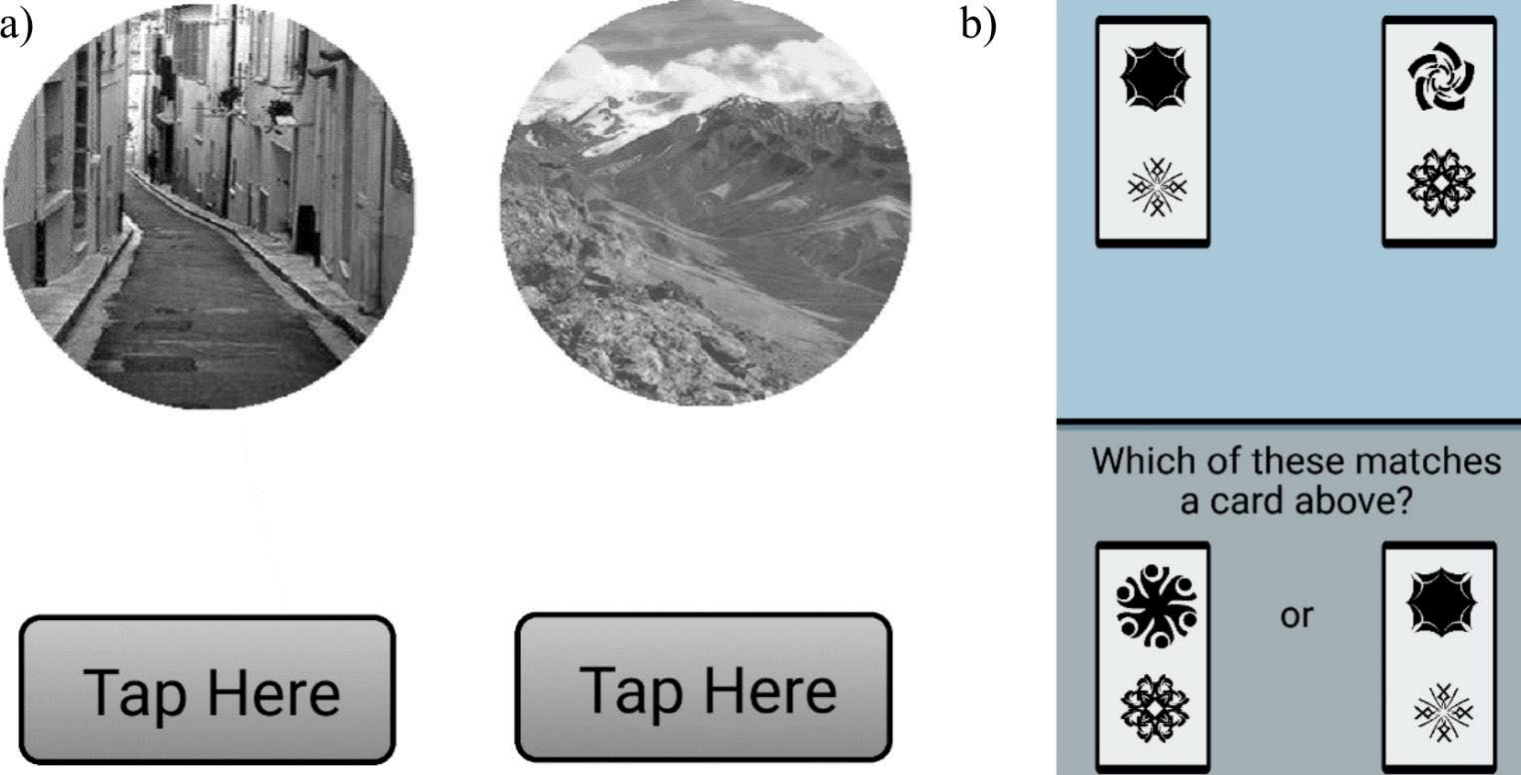
(a) Go/No-Go cognitive test on study phone screen. Participants are asked to tap the button upon seeing images of a city, but to not tap for mountains. (b) Symbol Search on study phone screen. Participants choose a card at the bottom of the screen that matches one on top

The two cognitive tests administered at the end of each EMA survey were the “Go/No-Go” and “Symbol Search” tasks. In the Go/No-Go task, a test of sustained attention ability (Fortenbaugh et al., 2015), participants were shown pictures of a city or mountain (Fig. 1a)., They were asked to tap an onscreen button as quickly as possible when a city was shown, and to withhold a response (i.e., to let the app automatically advance to the next image) when a mountain was presented. In each trial, participants were presented with 75 images, 8 of which were mountains and the rest cities. Each trial lasted approximately one minute. Numerous outcome measures can be calculated for the Go/No-Go task but the primary metric used is d’ (d-prime), which assesses the ability to discriminate between the cities and mountains. d’ is calculated for each trial with a signal detection approach based on the difference in the number of targets (mountains) a participant correctly withheld a response to, and the number of non-targets (cities) the participant incorrectly withheld a response to (Fortenbaugh et al., 2015). A secondary metric computed for the Go/No-Go tasks was criterion, which captures impulsivity when responding (Fortenbaugh et al., 2015). Criterion was used to examine the extent to which impulsivity may have impacted performance assessed with d’, and was calculated alongside d’ with a signal detection approach (Fortenbaugh et al., 2015). To attain an approximation of sustained attention ability over a whole day, d’ scores obtained over the same day were averaged. Averages were calculated at the daily level to match the temporal density of the whole day workload measure, which was administered once daily in the evening.

In the Symbol Search task, an assessment of visual-spatial attention and processing speed (Sliwinski et al., 2018), participants were presented with two cards at the top and bottom of the phone screen (Fig. 1b). Each card has two symbols on it. Participants were asked to press the card at the bottom of the screen that has symbols matching one of the cards at the top as quickly as possible, for twenty trials. This task typically lasted about 45 s. The primary performance metric used is the median reaction time for correct trials (Sliwinski et al., 2018), computed in milliseconds. The median reaction time scores for all Symbol Search trials in a day were averaged as an indicator of processing speed for each day.

Our third assessment of cognitive functioning was through paradata, specifically participants’ response times in EMA surveys. Paradata are data about survey behaviors collected automatically alongside survey responses (e.g. response time, keystrokes, etc.) (McClain et al., 2019). With timed cognitive tasks of minimal difficulty (e.g. classifying words as vegetables or animals) where there is a correct answer, response times serve as measures of processing speed (Kyllonen & Zu, 2016). Even though the association between cognition and response times on survey items without accuracy or time requirements (e.g. providing ratings of mood) is not widely established, evidence suggests that survey response times may capture cognitive functions related to processing speed (Roque et al., 2020). Survey response times were included in this study for exploratory purposes. Response times for each EMA survey item were automatically recorded in seconds, and log transformed to normalize their distribution. The mean of these log transformed values across all EMA survey items administered over the course of each day was calculated to represent the person’s average response time for the day.

To aid in interpretation, the Symbol Search score was reverse coded so that higher scores were associated with better performance when it was being compared to other measures (i.e. in correlations and structural equation modeling). In its raw forms, greater seconds (Symbol Search) to complete a task are associated with slower processing speed. This contrasts with sustained attention ability as measured by d’, where a higher score is indicative of better performance.

### Statistical analyses

#### Preliminary analyses

In initial analyses, we examined demographic information of participants, summary statistics for their cognitive performance measures, and their compliance with EMA surveys, to characterize our study sample. Additionally, concurrent within-person correlations and between-person correlations among the daily measures were calculated.

Reliabilities of study measures were examined. To assess the between-person reliability of the cognitive performance metrics, reliability was calculated with the formula BP Reliability = Var(BP)/(Var(BP) + Var(WP)/n), where Var(BP) is between person variance, Var(WP) is within-person variance, and *n* is the number of measurement occasions (Raykov & Marcoulides, 2006). Within-person reliability of the cognitive performance metrics was computed with the formula WP Reliability = Var(WP_occasion_)/(Var(WP_occasion_) + Var(WP_trial_)/i) (Cranford et al., 2006), where Var(WP_occasion_) represents the variance of the daily latent average of a cognitive performance metric, Var(WP_trial_) is the variance of the cognitive performance metrics within a day, and *i* is the number of measures in a day. Reliability of the whole day workload measure (six items) was assessed by calculating McDonald’s omega for the within and between-person levels based on multilevel confirmatory factor analysis procedures (Geldhof et al., 2014).

Prior to running the primary statistical models, the cognitive test scores were detrended as appropriate to address practice effects, which if unaccounted for can potentially result in spurious associations (Sliwinski et al., 2006). For each of the three cognitive measures (Symbol Search, Go/No-Go, question response times) we plotted the mean scores across study days to determine if a temporal trend was present (Sliwinski et al., 2006). To remove temporal trends, we estimated curvilinear regression models separately for each individual, where a given cognitive measure was regressed on linear and quadratic terms of “study day” (e.g. day count from the beginning of the study). The resulting regression residuals were added to an individual’s mean score across all measurement occasions, and this detrended cognitive measure was used in the primary analyses.

#### Primary analyses

We used dynamic structural equation modeling (DSEM; also called multilevel time series analysis) in M*plus* version 8.8 (Muthén & Muthén, 1998) to examine effects of whole day workload on next day cognition. DSEM is an extension of multilevel modeling that accounts for the non-independence of observations (multiple days nested in individuals) and allows for the estimation of lagged relationships across days in a latent variable framework (Asparouhov et al., 2018). The three (detrended) cognitive measures served as dependent variables in separate models. In all models, within-person centered workload from the day prior to the cognitive assessment was entered as a predictor at the within-person level. Self-reported sleep quality for the night between the report of workload and the subsequent day of cognitive assessments was included as a within-person centered covariate. We included autoregressive (i.e., lagged) effects of the cognitive scores to test if within-person variation in prior-day workload predicted cognitive performance after controlling for cognitive performance on the previous day (i.e. Granger causality) (Seth, 2007). A random intercept of the cognitive measure (dependent variable) was specified to account for non-independence of observations from the same individual (Preacher et al., 2010).

Several modeling choices were made to reduce DSEM complexity. Data we analyzed had a sample size of n = 56 and up to 14 observations per individual, both of which were relatively low compared to sizes of datasets for which DSEM is typically applied (Schultzberg & Muthén, 2018). The sample size needed for a particular DSEM depends on a number of factors, including the type of model specified and the random coefficients in the model (Schultzberg & Muthén, 2018). Generally, more complex DSEMs require larger sample sizes and numbers of observations per individual. The average of daily cognitive measures was used to avoid additional parameters that would have resulted from modeling daily means as a latent average. Results of both random intercept (simplest) and random slope (for the relationship between workload and cognitive performance) versions of models are presented. We individually detrended the cognitive performance measures prior to entering them into the models, to avoid the use of additional parameters that would have been required when accounting for temporal effects directly in DSEM (e.g. by adding study day and its squared version as covariates).

One popular approach to making causal inferences is analysis of longitudinal observational data that controls for confounds and models lagged relationships, and our use of a Granger causal model falls under this umbrella (Zyphur et al., 2020). Randomized controlled trials may often be considered optimal to control for potential confounders and infer causality, but they can often be difficult and costly to implement (Marinescu et al., 2018). When the conditions of Granger causality are met, one can be confident that one variable temporally precedes another (Granger, 1969), providing some (not absolute) evidence of a causal relationship between the variables of interest.

The DSEM implementation in M*plus* software is based on Bayesian parameter estimation using a Markov chain Monte Carlo (MCMC) algorithm, which accommodates missing values (due to missed prompts on some days) provided that they are missing at random (MAR). The Potential Scale Reduction statistic, a metric assessing the degree of similarity between MCMC chains (Gelman et al., 2015), was used to decide on the number of MCMC iterations needed for model convergence. We present regression estimates along with 95% credible intervals, which were used to determine statistical significance (these can be interpreted analogous to 95% confidence intervals) (Lu et al., 2012).

## Results

### Preliminary analyses

#### Demographics

Analyses were conducted on data from 56 workers who primarily worked full-time and had an average age of 39.4 (SD = 12.8) years, as shown in Table 1. About half (55%) of the sample were women. The largest ethnicity groups in the sample were White (41%) and Latino/x (27%), the biggest education groups were Bachelor’s degree (38%) and graduate degree (25%), and most frequent annual household income groups were ≥$100,000 (30%) and <$50,000 (25%). In terms of EMA survey completion, the final dataset included 4,051 observations from 872 days. Across the 872 days, the mean and standard deviation (SD, in parentheses) for the study variables was 6.58 (3.5) for hours worked on workdays, 43.86 (15.05) for whole day workload ratings on a scale of 0 (low workload) to 100, and 52.87 (24.84) for sleep quality on a scale of 0 (low sleep quality) to 100, respectively. The within and between-person SD for workload was 8.38 and 13.03 respectively. Reports of type of vocation were not required. Among participants that did report their occupation, a broad range of occupations were indicated including housekeeper, nurse, physician, lawyer, cashier, teacher, and law enforcement. Across all participants, ≥ 4 EMA surveys were completed on 83% of all data collection days. On average, participants had 12 days with at least four EMA surveys completed. The median EMA completion rate was 92%.

**Table 1:** Demographics for workload and cognitive performance study sample (n = 56)

Characteristic	n	Mean (SD) or Percentage (%)
Age (years)	56	39.4 (12.8)
Gender		
Male	25	45%
Female	31	55%
Ethnicity		
White	23	41%
Latino/x	15	27%
African American	8	14%
Multi-ethnic	4	7%
Other	6	11%
Employment status		
Full-time	44	79%
Part-time	12	21%
Education		
High school grad or less	7	13%
Some college, no degree	10	18%
Associate’s degree	4	7%
Bachelor’s degree	21	38%
Graduate degree	14	25%
Annual household income		
<$50,000	14	25%
$50,000-$99,999	12	21%
≥$100,000	17	30%
Don’t wish to provide	10	18%
Don’t know	3	5%

#### Descriptive statistics of cognitive measures

The table in Appendix B provides summary statistics for the cognitive performance metrics, both for all daily observations pooled together (n = 759) and for person-level averages (n = 56). The statistics from the person-level averages weigh all participants equally, while statistics for daily observations place greater weight on participants that completed more EMA surveys, and show the full range of performances across all surveys taken. Between-person standard deviations for the cognitive measures were 0.38 for the Symbol Search, 0.53 for the Go/No- Go (sustained attention), and 0.32 for log transformed mean response times. Within-person standard deviations for the cognitive measures were 0.16 for the Symbol Search, 0.33 for the Go/No- Go (sustained attention), and 0.13 for log transformed mean response times.

#### Concurrent within-person correlations

The between-person (above diagonal) and within-person (below diagonal) correlations of the study measures are shown in Table 2. Note that the within-person correlations were for averages of measures taken on the same day. No significant association was found between processing speed and sustained attention (p = .351). Processing speed and logged question response time were moderately intercorrelated (r=-.30, p < .001). A small association was evident between sustained attention ability and question response time (r = .11, p = .002) (Cohen, 2013). Whole day workload was not correlated with processing speed or sustained attention on the same day, whereas it had a small positive correlation with same-day question response time (r = .08, p = .022). Impulsivity when completing the sustained attention test was moderately correlated with same day processing speed (r = .26, p < .001), sustained attention (r=-.33, p < .001), and question response time (r=-.34, p < .001). In terms of the between-person level, a large correlation was seen between processing speed and question response times (r=-.65, p < .001). At level 2, impulsivity on the sustained attention task as measured by criterion had a large correlation with EMA response times (r=-.63, p < .001) and moderate associations with processing speed (r = .40, p = .002) and sustained attention ability (r=-.29, p = .03).

**Table 2:** Within-person intercorrelations between same day cognitive performance, workload, and sleep quality

	Processing speed^a^	Sustained attention	Logged question response time	Whole day workload	Sleep quality	Impuls-ivity
**Processing speed** ^**a**^	-	0.18, p = .178 (B)	-0.65, p < .001 (B)	0.10, p = .467 (B)	-0.19, p = .162 (B)	0.40, p = .002 (B)
**Sustained attention**	0.03, p = .351 (W)	-	0.18, p = .183 (B)	-0.09, p = .467 (B)	0.04, p = .771 (B)	-0.29, p = .03 (B)
**Logged question response time**	-0.30, p < .001 (W)	0.11, p = .002 (W)	-	-0.08, p = .550 (B)	0.10, p = .444 (B)	-0.63, p < .001 (B)
**Whole day workload**	− 0.03, p = .359 (W)	− 0.06, p = .133 (W)	0.08, p = .022 (W)	-	-0.17, p = .208 (B)	-0.16, p = .252 (B)
**Sleep quality**	− 0.05, p = .211 (W)	0.02, p = .544 (W)	− 0.03, p = .492 (W)	− 0.07, p = .097 (W)	-	-0.13, p = .358 (B)
**Impulsivity (criterion)**	0.26, p < .001 (W)	-0.33, p < .001 (W)	-0.34, p < .001 (W)	0.00, p = .964 (W)	0.06, p = .106 (W)	-

^a^To improve interpretability, processing speed was reverse coded so that higher values indicated better processing speed.

(B): between-person correlation; (W): within-person correlation.

#### Reliabilities

The within-person reliabilities of the cognitive performance metrics (assuming 6 measurement occasions in a day) were as follows: 0.57 for the processing speed measure, 0.26 for sustained attention, 0.72 for response times, and 0.46 for impulsivity. Between-person reliabilities for 14 days of measures were 0.96 for processing speed, 0.90 for sustained attention, 0.94 for response times, and 0.93 for impulsivity. For the whole day workload measure, McDonald’s omega was 0.69 at the within-person level, and 0.62 at the between-person level.

#### Longitudinal change in cognitive performance

**Fig. 2:**
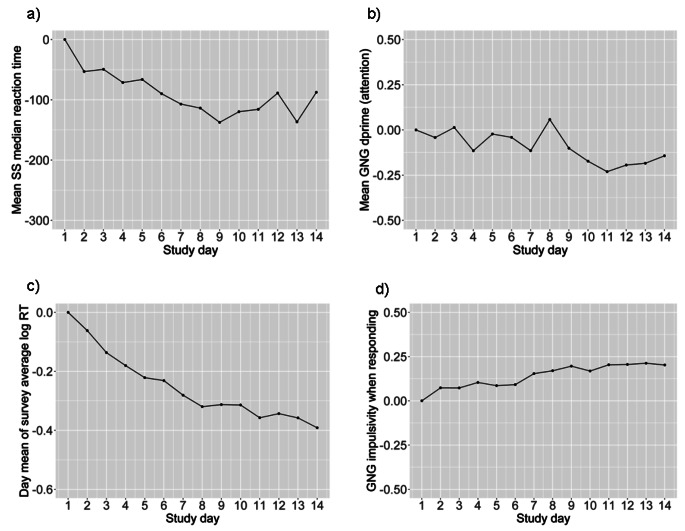
a) Mean Symbol Search (SS) median reaction time (centered by day 1 value) in milliseconds, and study day. Lower reaction time indicates faster processing speed. b) Mean Go No Go d’ (centered by day 1 value), and study day. Higher d’ indicates better sustained attention ability. c) Mean average of log of response times (RT) (centered by day 1 value), and study day. d) Mean Go No Go criterion (centered by day 1 value), and study day. Higher criterion indicates greater impulsivity when responding

Change in cognitive performance by study day appeared non-trivial (Fig. 2). Thus, detrending was applied to all three cognitive performance measures prior to their inclusion in DSEM models. Note that the mean sustained attention ability (d’) decreased over time, which was unexpected since practice effects typically result in improved performance. In post hoc analyses, we examined the possibility that this effect might have occurred due to an increase in impulsivity in responding, as indicated by increasing values on the *criterion* measure in the Go/No-Go task. As shown in Fig. 2d, we found that this was in fact the case, suggesting that the decreasing sustained ability scores may have been related to increasing impulsivity over time.

#### Primary analyses

With use of Bayesian parameter estimation in Mplus, smaller DIC values are indicative of better model fit (Muthén, 2010). For Mode1 1 (processing speed), the random intercept model had a deviance (DIC) of 14105.50 while the random slope model had a DIC of 14057.99. For Model 2 (sustained attention ability), the DIC values for the random intercept and slope models were 15245.36 and 15246.04, respectively. For Model 3 (log response times), the DICs for the random intercept and slope models were 13596.26 and 13569.93, respectively.

In the random intercept model, whole day workload, our focal predictor, had a significant lagged effect on processing speed on the next day (standardized estimate=-0.1, 95% CI -0.18 to -0.01); no significant lagged effects of workload were evident for sustained attention or question response time (Table 3). Put another way, for every one within-person standard deviation increase in whole day workload, processing speed decreased by 0.1 of a standard deviation on the next day. Taking into account the SD values, a 13-point increase in whole day workload (scale of 0 to 100) relative to a person’s average was associated with an increase in the next day average of the median response time on processing speed trials by 0.1*0.16 = 0.016 s, indicative of slower performance. With rescaling, it can equivalently be said that a 10-point increase in whole day workload relative to a person’s average was associated with an increase in the next day average of the median response time on processing speed trials by 0.012 s. When a random slope was specified, whole day workload did not have a significant effect on next day processing speed (standardized estimate= -0.07, 95% CI -0.15 to 0.01). Sleep quality was found to have a significant association with next day EMA response times in both the random intercept (standardized estimate=-0.1, 95% CI, 95% CI -0.18 to -0.02) and random slope models (standardized estimate=-0.1, 95% CI -0.18 to -0.03), but not next day processing speed or sustained attention. At the between person level, neither whole day workload or sleep quality had significant relationships with the averages of the different cognitive performance metrics (Table 4).

**Table 3:** Standardized within-person parameter estimates from mixed effects models with cognitive performance metrics regressed on whole day workload from the day prior at level 1. For each model, one version tested had a random intercept only. The other version had a random intercept and random slope for the relationship between cognitive performance and whole day workload

	Random intercept only			Random intercept and random slope		
	Stand. Estimate	95% CI Lower	95% CI Upper	Stand. Estimate	95% CI Lower	95% CI Upper
Model 1: Relationship between **Symbol Search (processing speed)**^a^ workload on the day prior						
**Whole Day Workload**	-0.1*	-0.18	-0.01	-0.07	-0.15	0.01
Symbol Search (autoregressive term)	0.06	-0.02	0.15	0.05	-0.04	0.14
Sleep quality of night workload recorded	0	-0.09	0.09	0	-0.08	0.08
Symbol Search residual variance	0.98*	0.95	1	0.82*	0.78	0.86
Model 2: Relationship between **Go No Go (sustained attention)** and workload on the day prior						
**Whole Day Workload**	-0.04	-0.12	0.04	-0.04	-0.12	0.04
Go No Go (autoregressive term)	-0.1*	-0.19	-0.02	-0.12*	-0.2	-0.03
Sleep quality of night workload recorded	0.01	-0.08	0.09	0	-0.08	0.08
Go No Go residual variance	0.98*	0.96	1	0.83*	0.78	0.87
Model 3: Relationship between **average of log of response times** and workload on the day prior						
**Whole Day Workload**	-0.02	-0.1	0.06	-0.02	-0.1	0.06
Average of logged response times (autoregressive term)	0.11*	0.02	0.19	0.08	-0.01	0.16
Sleep quality of night workload recorded	-0.1*	-0.18	-0.02	-0.1*	-0.18	-0.03
Response Times residual variance	0.97*	0.94	0.99	0.81*	0.76	0.85

*CI does not contain 0, indicating statistical significance

^a^Symbol Search values were reversed so that higher values indicated better performance.

**Table 4:** Standardized between-person parameter estimates from mixed effects models with cognitive performance metrics regressed on whole day workload at level 2. For each model, one version tested had a random intercept only. The other version had a random intercept and random slope for the relationship between cognitive performance and whole day workload

	Random intercept only			Random intercept and random slope		
	Stand. Estimate	95% CI Lower	95% CI Upper	Stand. Estimate	95% CI Lower	95% CI Upper
Model 1: Relationship between **Symbol Search (processing speed)**^**a**^**average** and workload						
**Whole Day Workload**	0.07	-0.22	0.36	0.07	-0.22	0.36
Sleep quality	-0.2	-0.46	0.1	-0.19	-0.46	0.1
Symbol Search intercept	-3.91*	-6.06	-1.6	-3.82*	-5.97	-1.47
Symbol Search residual variance	0.93*	0.75	1	0.93*	0.76	1
Model 2: Relationship between **Go No Go (sustained attention) average** and workload						
**Whole Day Workload**	-0.04	-0.33	0.26	-0.03	-0.32	0.27
Sleep quality	0.03	-0.27	0.33	0.04	-0.26	0.32
Go No Go intercept	4.59*	2.17	6.68	4.39*	2.03	6.5
Go No Go residual variance	0.97*	0.83	1	0.97*	0.84	1
Model 3: Relationship between **average of log of response times average** and workload						
**Whole Day Workload**	-0.05	-0.33	0.25	-0.06	-0.34	0.23
Sleep quality	0.13	-0.17	0.41	0.13	-0.17	0.41
Response Times intercept	3.02*	0.79	5.06	3*	0.84	4.99
Response Times residual variance	0.95*	0.8	1	0.95*	0.8	1

*CI does not contain 0, indicating statistical significance

^a^Symbol Search values were reversed so that higher values indicated better performance.

## Discussion

In this intensive longitudinal study, a Granger causal relationship was observed between within-person whole day workload (person-mean centered whole day workload), and visual processing speed on the next day, after controlling for sleep quality, only for the random intercept but not the random slope model. Specifically, in the random intercept model, experiencing higher whole day workload was associated with slower next day processing speed, with a small effect size (standardized estimate =-0.10). Granger causal methods provide greater confidence that the relationship is potentially causal, because the association between the two was significant after controlling for the value of the dependent variable on the day prior (Seth, 2007). The finding of a significant relationship between these variables contrasts with the null relationship found in a prior study, which was inclusive of both workers and non-workers, and where practice effects on the cognitive tests were not accounted for (Hernandez et al., 2021a, b). The model allowing for a random slope had better fit (i.e. a lower DIC) and made the more reasonable assumption that the relationship between workload and next day processing speed varies by person. In this model, the relationship between whole day workload and processing speed was not significant but demonstrated a tendency towards the expected effect. This may have in part been due to the greater model complexity, small sample size, and associated lower statistical power for detecting significant effects when allowing for regression slopes to be random.

Note that more sophisticated methods of modeling causality in observational studies exist that were not used here, such as the general cross-lagged panel model (GCLM). GLCM allows modeling changes in cross-lagged or autoregressive terms over time, instead of the approach taken in this paper of assuming that these parameters were constant across time. With a GCLM, it is also possible to investigate bidirectional lagged relationships and to distinguish between short and long-term effects (Zyphur et al., 2020). In the models examined here however, only the path from workload to next day cognitive performance was tested, and no distinction was made between short and longer-term effects. Use of methods such as GCLM should be considered in future studies with larger sample sizes.

Within-person whole day workload was not found to have a significant association with next day question response time or sustained attention ability. Given the moderate within-person correlation between processing speed and question response time (r=-.30), survey question response time appeared to also represent processing speed to an extent. We therefore expected response time to have a significant relationship with whole day workload on the day prior like with processing speed, but this relationship was not what we observed. Response times may not only be impacted by processing speed, but by other factors such as response caution (Kyllonen & Zu, 2016). Higher whole day workload may result in poorer next day processing speed and lower response caution, which combined can appear as no effect on response times. We had also anticipated that higher whole day workload would be associated with decreased sustained attention ability the next day, as prior work has found long working hours to be associated with impaired attention (Flinn & Armstrong, 2011). No association between the two was seen however in this study, perhaps because of more limited exposure to whole day workload. In the prior study, workers were exposed to long working hours (Flinn & Armstrong, 2011), while for this study we found that the average number of work hours reported on workdays was 6.58.

In terms of potentially counterintuitive results, processing speed and question response time demonstrated the expected practice effects where performance improved over time, but sustained attention ability was found to decrease by study day. A possible reason for the decreasing mean sustained attention ability scores is that participants may have adopted a more impulsive response strategy over time (i.e., with repeated administration of the same test over the course of the study), meaning they responded to the test faster but at the cost of performance. Supporting this assertion, we found that Go/No-Go criterion, a measure of the willingness to respond in the case of uncertainty (Fortenbaugh et al., 2015), increased over time (Fig. 2d), and that sustained attention ability and criterion had a negative correlation at the within-person level (r=-.33, p < .001). In follow up analyses, we conducted a multilevel linear growth model where both sustained attention ability and impulsivity were regressed on study day at level 1, and random slopes for both were specified. Sustained attention ability was found to decrease on average over time (standardized estimate=-0.15, 95% CI -0.20 to -0.07) while criterion increased on average over time (standardized estimate = 0.26, 95% CI 0.21 to 0.32). However, the random intercepts and growth rates were not significantly correlated between the two measures, which suggests that there was no pronounced relationship between decreasing sustained attention ability and increasing impulsivity over time.

Sleep quality was found to only have a relationship with next day response times, but not sustained attention ability or processing speed, which contrasts with findings from prior research (Alhola & Polo-Kantola, 2007; Kaliyaperumal et al., 2017). One possible explanation is that sustained attention/processing speed deficits may only occur with notable sleep deficits, whereas the mean sleep quality rating in our sample was 52.87 (SD 24.84) on a scale of 0 (low sleep quality) to 100. Thus, sleep quality in our sample may not have been as poor as in other samples such as nurses (Kaliyaperumal et al., 2017). Higher sleep quality may have been associated with faster next day survey response times, perhaps because survey response times require some aspect(s) of cognition more susceptible to poorer sleep quality.

It is difficult to compare the processing speed and sustained attention ability scores in the present sample with population norms because the number of trials used for tests, and the period over which tests were taken, were different compared to prior administrations. In a previous study, adults that were 40 years of age (mean age of our sample was 39), who completed a longer 299 trial version of the same sustained attention ability test (Go/No-Go) in a single trial, had a mean d’ score of approximately 3.25 (Fortenbaugh et al., 2015). This number is higher than the average of 2.31 found here, and suggests a lower mean sustained attention ability for our sample. Adults with a mean age of 47, who completed a shorter 12 trial version of the processing speed test (Symbol Search) in repeated trials over two days, had an average of 2700 (SD = 670) milliseconds in a prior study (Sliwinski et al., 2018). This is notably slower than the average of 1602.88 milliseconds in the present study, suggesting higher mean processing speed for our sample over an approximately two-week period. The prior administration of the sustained attention ability test was done in a single trial (Fortenbaugh et al., 2015), but in this study the Go No Go task was administered over approximately two weeks, at set times throughout the day that may have not always been conducive to concentration. Thus, the lower mean sustained attention ability in our sample may have been a consequence of a greater likelihood of being distracted when taking tests in the ambulatory assessment context, and may be a more ecologically valid reflection of people’s sustained attention ability in everyday life. In terms of processing speed, participants in the prior study completed Symbol Search tests in the ambulatory context over two days (Sliwinski et al., 2018). Here however, the Symbol Search test was completed over a much longer period, which allowed for more practice effects, and thus better mean performance.

Whole day workload is a measure that aligns with NIOSH’s paradigm shift to a more holistic worker well-being framework (Chari et al., 2018), and this study contributes to the emerging research on this construct. A prior study found within-person relationships between whole day workload and well-being measures such as stress, fatigue, and positive affect (Hernandez et al., 2021a, b). Here, we found that, in a random intercept model, greater whole day workload was associated with decreased processing speed the next day (after accounting for practice effects), suggesting that whole day workload may be relevant not only to well-being, but to work performance as well.

Our results suggested that just a single day of workload had a possible subtle impact on next day processing speed. Should future research corroborate this finding, there may be implications for strategic management of workers’ whole day workload for work performance optimization. One implication may be that cognitive performance may not only be impacted by a person’s average workload levels (e.g. average working hours) (Virtanen et al., 2009) or momentary workload (e.g. momentary physical load) (Fiľo & Janoušek, 2022), but it also may be affected by workload over the course of the previous day, suggesting that workload over each of these time frames would need to be considered. For instance, for high stakes meetings or challenging medical procedures, workers would likely prefer to be in an optimal cognitive state. One way administrators and/or other staff could help to optimize cognitive state could be by minimizing acute (single day) effects of workload, which may entail scheduling such that the day prior to these events is associated with lower workload. Alternatively, the onus may be on workers themselves to try to schedule their commitments in such a way that days when optimal performance is needed are preceded by lower whole day workload days. Addressing workload on the day prior might be one out of several components for promoting optimal cognitive performance, as the effects of average and momentary workload might also need to be considered.

### Limitations

Our study sample was comprised of workers with type 1 diabetes experiencing various stages of the COVID-19 pandemic, which may limit the generalizability of study findings. There may be subtle differences in cognition between those with T1D and the general population, such as mild to moderate deficits in mental speed and flexibility (Brands et al., 2005). Individuals with T1D may have complications such as visual acuity issues secondary to retinopathy, and fine motor difficulties secondary to peripheral neuropathy (Bloomgarden, 2005), that may impact performance on phone based cognitive tests. However, inclusion in the study from which data was analyzed required sufficient visual acuity and fine motor skills to complete study tasks, including the phone based cognitive tests. Thus, visual acuity and fine motor difficulties were likely not hindrances to completion of cognitive tasks for most participants, but the implication is that lower functioning individuals with T1D with such complications were not represented in the data.

Limitations of our statistical analyses should be noted. Firstly, the sample size of n = 56 and up to 14 observations per individual limited the degree of complexity with which we could specify DSEMs, and also limited the power to detect small between-person relationships and, with random slopes specified, small within-person associations. A larger sample of workers, and greater number of repeated measures, may be needed to allow testing of more complex models, and detection of smaller effects. Additionally, the within-person reliabilities of study measures could have been improved, as many were below 0.70. Within-person reliability of the daily sustained attention ability measure was particularly low, with a value of 0.26. Some have argued that universal application of the 0.70 standard to all measurement contexts may not be optimal, and that, for instance, more relaxed criteria may be reasonable for within-person reliability (Nezlek, 2017). One argument for this was that within-person reliability is often calculated in repeated measures studies, where the number of trials at each measurement occasion is kept minimal to reduce participant burden (Nezlek, 2017). Since a high number of trials at each measurement occasion is not always feasible, within-person reliability may then also be correspondingly lower.

Our results suggested that greater whole day workload preceded slower next day processing speed, but do not provide strong proof of a causal relationship. One way that the causality argument could be strengthened is through use of more complex models such as GCLM (described above) to investigate causality. The argument of causality may also be strengthened with a controlled experiment, where participants are randomly assigned to days of varying degrees of workload, and then asked to complete cognitive testing on the next day. The logistics of such a study may be difficult, but a controlled experiment could provide stronger evidence of a causal link between whole day workload and next day cognitive performance.

## Conclusion

Our results suggested that greater whole day workload compared to one’s average had a Granger causal relationship with decreased visual processing speed the next day, but not with sustained attention ability or question response time. The relationship between whole day workload and next day processing speed was significant after controlling for sleep quality and after detrending the processing speed scores for possible practice effects. It was not significant however and demonstrated only a tendency toward the expected effect when a random slope was specified for the relationship between whole day workload and processing speed. Whole day workload is more holistic compared to work specific workload measures, and our results contribute to the emerging evidence supporting whole day workload’s potential relevance to worker performance.

## Data Availability

The data that support the findings of this study are available from the corresponding author, R.H., upon reasonable request.

## Appendix A: study measures

**Table:**

Construct	Item(s)/Assessment Method	Scoring	Time
Sustained attention ability	Go No Go test	Calculated using a signal detection approach considering the frequencies of correct withholding of responses and incorrect withholding of responses	All survey times
Impulsivity when responding	Go No Go test	Calculated with the same signal detection approach described above	All survey times
Processing speed	Symbol Search Test	Greater processing speed indicated by a lower median response time in trials where symbols were accurately matched	All survey times
Unconfirmed aspects of cognition	Response time for each question recorded	N/A	All survey times
Whole day workload	Six NASA-TLX items ask about mental demand, physical demand, time pressure, effort, performance satisfaction, and frustration for activities over the whole day. The sum of the ratings indicates total workload.	0 to 100 sliding scale for each item	Last survey of day
Sleep quality	How well rested do you feel?	0 (Not at all rested) to 100 (Extremely rested)	First survey of day

## Appendix B: cognitive performance summary statistics

**Table:**

	All observations pooled together (n = 759)			Scores for each cluster averaged (n = 56)		
	Mean (SD)	Minimum	Maximum	Mean (SD)	Minimum	Maximum
**Processing speed** ^**a**^	1588.53(392.06)	743.60	3619.50	1602.88(356.23)	986.65	2689.34
**Sustained attention**	2.29(0.63)	− 0.20	3.97	2.31(0.50)	0.60	3.34
**Log of question response time (unconfirmed aspect of cognition)**	0.88(0.33)	0.03	2.05	1.05(0.30)	0.41	1.68

^a^Processing speed was not reverse coded here for greater ease of comparison with past processing speed scores.

## Code Availability

The code that support the findings of this study are available from the corresponding author, R.H., upon reasonable request.
